# Sweat Testing for the Detection of Methylone after Controlled Administrations in Humans

**DOI:** 10.3390/ijms24087395

**Published:** 2023-04-17

**Authors:** Alessandro Di Giorgi, Giorgia Sprega, Lourdes Poyatos, Esther Papaseit, Clara Pérez-Mañá, Annagiulia Di Trana, Maria Rosaria Varì, Francesco Paolo Busardò, Simona Pichini, Simona Zaami, Alfredo Fabrizio Lo Faro, Magí Farré

**Affiliations:** 1 Department of Excellence-Biomedical Sciences and Public Health, Università Politecnica delle Marche, 60121 Ancona, Italy; digiorgiale97@gmail.com (A.D.G.); giorgiasprega1996@gmail.com (G.S.); f.busardo@univpm.it (F.P.B.); fabriziolofaro09@gmail.com (A.F.L.F.); 2 Department of Clinical Pharmacology, Hospital Universitari Germans Trias i Pujol and Institut de Recerca Germans Trias i Pujol (HUGTiP-IGTP), 08916 Badalona, Spain; lpoyatos@igtp.cat (L.P.); epapaseit.germanstrias@gencat.cat (E.P.); cperezm.mn.ics@gencat.cat (C.P.-M.); mfarre.germanstrias@gencat.cat (M.F.); 3 Department of Pharmacology, Therapeutics and Toxicology and Department of Psychiatry and Forensic Medicine, Universitat Autònoma de Barcelona (UAB), 08193 Cerdanyola del Vallés, Spain; 4 National Centre on Addiction and Doping, Istituto Superiore di Sanità, 00161 Rome, Italy; annagiulia.ditrana@iss.it (A.D.T.); simona.pichini@iss.it (S.P.); 5 Department of Anatomical, Histological, Forensic, and Orthopedic Sciences, Università La Sapienza, 00161 Rome, Italy; simona.zaami@uniroma1.it

**Keywords:** methylone, sweat, LC-MS/MS, pharmacokinetics

## Abstract

The aim of this study was to determine the excretion of methylone and its metabolites in sweat following the ingestion of increasing controlled doses of 50, 100, 150 and 200 mg of methylone to twelve healthy volunteers involved in a clinical trial. Methylone and its metabolites 4-hydroxy-3-methoxy-N-methylcathinone (HMMC) and 3,4-methylenedioxycathinone (MDC) were analyzed in sweat patches by liquid chromatography–tandem mass spectrometry. Methylone and MDC were detected in sweat at 2 h and reached their highest accumulation (C_max_) at 24 h after the administration of 50, 100, 150 and 200 mg doses. In contrast, HMMC was not detectable at any time interval after each dose. Sweat proved to be a suitable matrix for methylone and its metabolites’ determination in clinical and toxicological studies, providing a concentration that reveals recent drug consumption.

## 1. Introduction

Synthetic cathinones constitute the most extensive class of synthetic stimulants, compounds belonging to the group of New Psychoactive Substances (NPS). Their structure is based on that of cathinone, a monoamine alkaloid found in the khat plant with psychoactive effects [1]. One of the most abused compounds of this class is 3,4-methylenedioxy-methcathinone (MDMC), more frequently known as methylone [2]. This drug often appears as a white powder [3]. The structure of methylone differs only from that of 3,4-methylenedioxy-methamphetamine (MDMA) for the ketone group in the β-position [4]. Consequently, these two compounds share a similar mechanism of action, which involves the enhancement of monoamine release (norepinephrine, dopamine and serotonin) and the inhibition of the dopamine transporter (DAT), the serotonin transporter (SERT) and the norepinephrine transporter (NET) [4,5,6].

As MDMA, methylone is mainly metabolized in the liver. The two major pathways are N-demethylation, which converts this drug to 3,4-methylenedioxycathinone (MDC), and O-demethylation, which is followed by O-methylation, resulting in 3,4-dihydroxy-N-methylcathinone (HHMC) and 4-hydroxy-3-methyoxy-N-methylcathinone (HMMC), respectively.

The pharmacological effects of methylone in humans resemble those of MDMA, including altered perception (such as changes in light, different bodily sensations), empathy, increased sociability, stimulation and euphoria [7]. Similar to other synthetic cathinones, methylone use has been shown to be related to consumer fatalities [1]. In this context, several cases of death are reported in the literature. A 23-year-old man was transported to the hospital with a body temperature of 41 °C and aggressive behavior; his death occurred after 3 h. Since high concentrations of methylone were detected in his biological matrices, the cause of death was attributed to this drug [8]. In another case, methylone was ingested during a party with friends [9]. 

Sweat is an alternative matrix useful for drug testing and monitoring; it is generally collected by applying an adhesive patch to different parts of the body (back, shoulders and chest). The exact mechanism of excretion of drugs in sweat is not still fully explained, but there are several proposed mechanisms, which include passive diffusion from the blood into the sweat glands. Specifically, since sweat is more acidic than blood (pH 6.3 and 7.4, respectively), basic drugs diffuse into sweat through a concentration gradient [10]. An alternative mechanism involves the diffusion of drugs through the stratum corneum to the skin surface, dissolving in sweat [11]. Moreover, the reabsorption of MDMA from the skin was reported [12], and this may result in the continued presence of drugs on the skin surface also within time windows not covered by other biological matrices such as blood or urine. Several analytical methods for the determination of methylone in other matrices have been reported in the literature, such as in plasma and serum [2], urine [13], oral fluid [14] and hair [15]. However, to the best of our knowledge, no research in sweat has been yet published, nor pharmacokinetic studies involving this matrix.

The aims of this study were as follows:

- to develop and validate an LC-MS/MS method for the determination of methylone and its metabolites, MDC and HMMC, in sweat;
- to investigate the excretion of methylone, MDC and HMMC in sweat after the ingestion of increasing controlled doses (50, 100, 150 and 200 mg) to 12 healthy volunteers involved in a clinical trial.

## 2. Results

**Figure 1:**
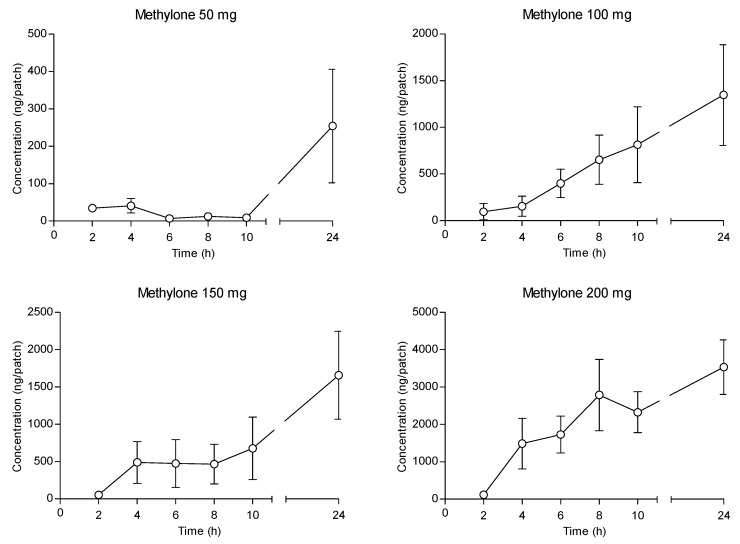
Mean concentration–time profiles of methylone contained in sweat patches following increasing controlled administrations (50, 100, 150 and 200 mg).

Figure 1 shows methylone’s mean concentration–time profile for each administered dose (50, 100, 150 and 200 mg). The drug was detected in sweat at 2 h after each dose at different concentrations (34.3, 96.0, 52.8 and 115.0 ng/patch following 50, 100, 150 and 200 mg doses, respectively). It has to be underlined that sweat concentrations are not temporal fractions, but reflect the accumulation of the parent drug and metabolites.

In this context, an interesting concentration–time profile was observed after each dose. In particular, the administration of 50 mg methylone presented a first peak value at 4 h (mean: 40.8 ng/patch), with no further release, leading to a mean concentration of 6.7, 12.3 and 9.0 ng/patch at 6, 8 and 10 h post-administration, respectively. Finally, methylone reached the mean value of 254.2 ng/patch at 24 h, representing drug accumulation in 24 h. Inter-subject variability was large, with a coefficient of variation, CV (%), ranging from 19% (at 8 and 10 h) to 60% (at 24 h). 

The administration of 100 mg methylone showed progressive drug excretion resulting in mean sweat concentrations of 96.0, 154.6, 398.9 and 651.9 ng/patch at 2, 4, 6 and 8 h post-administration. Then, concentrations rapidly increased to 813.7 ng/patch at 10 h. Similarly to what occurred with the 50 mg dose, the highest drug accumulation was observed at 24 h (mean: 1346.8 ng/patch, 5.3-fold higher than that obtained after 50 mg methylone). However, this subject showed the highest CV (%) values, ranging from 85% (at 6 h) to 201% (at 2 h).

Following 150 mg methylone dose administration, sweat concentrations remained constant between 4, 6 and 8 h, with values of 487.9, 474.6 and 466.3 ng/patch, respectively. Then, an increase to 676.1 ng/patch was observed at 10 h, and the highest accumulation at 24 h (1655.7 ng/patch). Inter-subject variability was large, between 36% (at 24 h) and 67% (at 6 h).

A different excretion profile was observed after the administration of the 200 mg dose. A concentration of 115.0 ng/patch methylone was detected at 2 h, and then the concentration rapidly increased to 1486.6 at 4 h. Between 4 and 6 h post-treatment, the concentration slightly increased to 1727.7 ng/patch, followed by a further rapid increase to 2784.6 at 8 h. At 10 h, methylone showed a slight decrease to 2324.6 ng/patch. Similarly to each administration, the highest drug accumulation in sweat was observed at 24 h, with a mean value of 3530.6 ng/patch. Inter-subject variability was lower than that observed for lower doses, with values that ranged between 21% and 51%.

**Figure 2:**
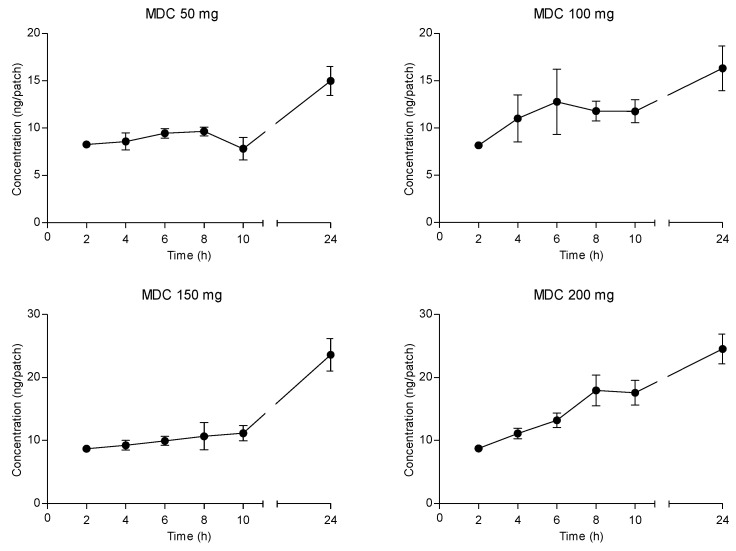
Concentration–time profile of metabolite MDC contained in sweat patches following increased controlled methylone administrations (50, 100, 150 and 200 mg).

Sweat specimens were also analyzed for methylone metabolites. HMMC, the major plasma metabolite, was not detected after each administered dose and the only metabolite detected was MDC (Figure 2). This compound was detected at 2 h with values of 8.3, 8.2, 8.7 and 8.8 ng/patch after the administration of 50, 100, 150 and 200 mg methylone. Then, a slight increase was observed between 2 and 8 h following the lowest dose, prior to a decrease at 10 h. Conversely, a more rapid increase was noticed between 2 and 6 h after the 100 mg dose, followed by constant values between 6 and 10 h. A slight progressive increase was observed between 2 and 10 h when 150 mg methylone was administered. A different profile was noticed after the 200 mg dose. MDC sweat concentrations rapidly increased between 2 and 8 h and remained constant between 8 and 10 h. For each administered dose, a rapid increase was observed at 10 h until reaching the highest metabolite accumulation of 15.0, 16.6, 23.6 and 24.5 ng/patch at 24 h after the 50, 100, 150 and 200 mg doses, respectively.

**Figure 3:**
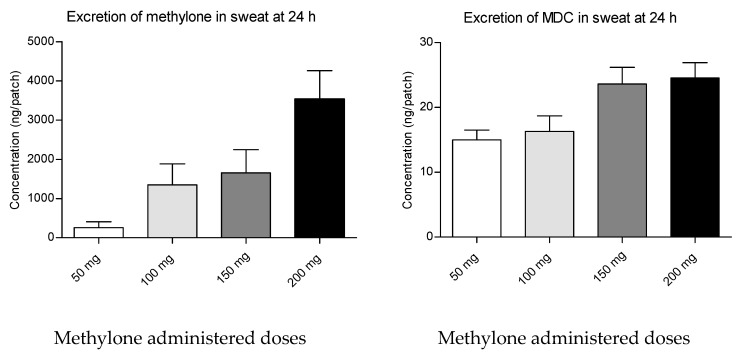
The 24 h excretion of methylone and MDC in sweat following increased controlled methylone administrations (50, 100, 150 and 200 mg).

A graph comparing the administered methylone doses and the accumulation of the parent drug and its metabolite at 24 h is shown in Figure 3.

## 3. Discussion

For the first time, sweat patches were used to monitor methylone excretion in human sweat for 24 h after drug administration. Drugs are mainly incorporated into this matrix through passive diffusion, due to a concentration gradient that allows the diffusion of the free fraction of the drug from plasma to sweat [10,16]. Moreover, sweat has a pH of 6.3 in normal conditions; thus, it is more acidic than blood [17]. Methylone is a basic drug with a pK_a_ around 7.96; consequently, this drug should convert to its ionized form and accumulate in sweat [10,17]. In this context, lipophilicity is another physical–chemical parameter that can suggest the presence of drugs in this matrix. Indeed, parent compounds crossing membranes can be detectable at higher concentrations than their more polar metabolites [12,18,19].

Similarly to oral fluid, the Henderson–Hasselbach equation allows the calculation of the excretion of a basic drug also in sweat, using the pH of blood and sweat, and the pK_a_ of the drug [10]. In particular, the theoretical sweat/plasma ratio for methylone should be around 11.2. However, we could not verify this theoretical calculation since, in our study, we applied sweat patches that did not allow the calculation of the volume of collected sweat. Moreover, the loss of water during wear could lead to an effect on these measures. 

Methylone was detectable in sweat at 2 h after the administration of all doses, corresponding to its peak concentration in plasma and oral fluid, and reached the highest concentration in sweat at 24 h, corresponding to the lowest methylone concentrations in plasma and oral fluid [2,20]. As expected, the main compound detected in sweat was the parent drug; the metabolite MDC was found at lower concentrations, while HMMC was not detected at any time interval. This is probably due to the more hydrophilic nature of the metabolites compared to methylone. Moreover, the presence of a secondary amine and a hydroxyl group lead HMMC to be more polar than MDC, which has a primary amine but also a dioxolane heterocycle. This is in agreement with a previous study on the sweat testing of MDMA following controlled administration. Indeed, its primary hydrophilic metabolite HMMA was found only in traces in the sweat patches of administered volunteers [12]. A constant MDC concentration was observed between 8 and 10 h following the administration of 100, 150 and 200 mg methylone doses. We can hypothesize that the resting period may affect the perspiration rate and the accumulation of the drug in sweat. 

Several studies demonstrated that the concentrations of drugs in sweat can decrease due to skin reabsorption, as observed for diazepam, MBDB, MDMA and cocaine [21,22,23]. This phenomenon could have explained the decrease in methylone concentrations noticed in some cases in this research. However, a confirmation study on skin reabsorption was not carried out.

Overall, the sweat patch allowed us to monitor methylone accumulation in sweat during the 24 h after administration. Since the local ethical committee’s permission was provided for a 24 h study, we could not investigate methylone excretion in sweat over a longer period of time. However, based on a previous study on MDMA, which was detectable up to a week after a single administration [24], we can hypothesize that this drug can be excreted in sweat for more than 24 h. Methylone concentrations at 24 h and the ratio between the area of the patch and the body surface area were used to approximate the total amount of methylone excreted in sweat. The mean value of methylone excreted during 24 h post-administration was 0.31, 3.85, 2.02 and 4.3 mg following 50, 100, 150 and 200 mg doses, respectively. These values are equivalent to 0.62%, 3.85%, 1.35% and 2.15% of 50, 100, 150 and 200 mg administered, respectively, considering equal sweating at each body location.

The large inter-subject variability observed in this study can be justified by the different perspiration rates and volumes of sweat between the individuals who received the same methylone dose. These differences were reported also for MDMA in a previous research study [12]. Moreover, the pharmacological activity of methylone regarding body temperature regulation contributed to the enhanced inter-subject variability. Another factor that contributed to the inter-subject variability was the location of the patch. In this context, a study comparing cocaine and metabolite concentrations in sweat patches applied on the back and on the shoulders found eight-fold higher levels on the back than on the shoulders [17]. For this reason, the sweat patches in this study were applied on the back, to determine the highest drug concentrations in sweat.

## 4. Materials and Methods

### 4.1. Subjects and Study Design

In total, 14 male volunteers were included in a cross-over, randomized, double-blind and placebo-controlled study at the Hospital Universitari Germans Trias i Pujol, Institut d’Investigació en Ciències de la Salut Germans Trias i Pujol, in Badalona (Spain). All volunteers were recreational users of drugs such as MDMA, amphetamines, synthetic cathinones and cocaine. Each participant received routine laboratory tests, a general physical examination, urinalysis and a 12-lead electrocardiogram. Table 1 presents the characteristic of the participants. All volunteers claimed to have consumed MDMA in the range of 5–100 times within their lives, with a mean value of 24 times. All subjects gave written informed consent before their inclusion in the research and were economically compensated for the inconveniences caused by their participation in the study. The study was carried out in accordance with the Declaration of Helsinki, approved by the local ethical committee for human research (CEIC-HuGTiP, ref. PI-19-082). 

Volunteers were divided into four groups and underwent three administration sessions, divided by a washout time of 5–7 days. Each participant was administered single oral doses of the placebo (dextromaltose), 50, 100, 150 or 200 mg methylone in each session. The selected methylone doses were based on previous observational studies reporting methylone and mephedrone’s effects. The World Health Organization reported 60–120 mg methylone doses as “threshold”, 100–150 mg as “light”, 100–250 mg as “common”, 160–270 mg as “strong” and >250 mg as “very strong” [25]. In a previous observational study, oral doses of 100 mg (*n* = 1), 150 mg (*n* = 2; 1 male and 1 female), 200 mg (*n* = 4; 2 male and 2 female) and 300 mg (*n* = 1; male) were well tolerated [26]. Moreover, in a controlled trial with mephedrone, we carried out a dose-finding pilot study with doses of 50, 100, 150 and 200 mg mephedrone and 100 mg MDMA. The highest methylone dose produced similar effects to that observed following the administration of 100 mg MDMA [27,28]. Based on the above-reported studies, we decided to administer well-tolerated methylone doses and the one (200 mg) producing effects similar to those obtained with 100 mg MDMA.

The administration of higher methylone doses was performed after lower ones. Moreover, good tolerability was noticed after each dose. Subjects were required not to consume any drug of abuse during the period of the study. Nonetheless, a urine drug test preceded each experimental session to confirm their abstinence. In particular, the drugs screened were barbiturate, cocaine, amphetamine, benzodiazepine, morphine, MDMA, methadone, methamphetamine, tetrahydrocannabinol and tricyclic antidepressants (Drug-Screen Multi 10TD Test [Multi-Line], Nal von Minden, Germany). For each class of compounds tested, all participants tested negative. Soft gelatin capsules of placebo and methylone were prepared by the Pharmacy Service of Hospital Universitari Germans Trias i Pujol and administered with 200 mL of tap water in a fasting state.

### 4.2. Chemicals

Methylone, MDC and HMMC were supplied by Cerilliant (Round Rock, TX, USA). Internal standard (IS), methylone-d_3_, was purchased from Cayman Chemical (Ann Arbor, MI, USA). Standards were stored at −20 °C until analysis. LC-MS-grade water, methanol, acetonitrile, formic acid and ethyl acetate were obtained from Carlo Erba (Cornaredo, Italy). Ammonium hydroxide (25% purity) and hydrochloric acid (37% purity) were supplied by Honeywell Fluka™ (Morristown, NJ, USA).

### 4.3. Sweat Sample Collection

Six patches were located on the back of each volunteer after the skin was cleaned with a 70% 2-propanol cotton swab and removed at 2, 4, 6, 8, 10 and 24 h after methylone/placebo administration. This was performed by pulling the adhesive edge, taking care not to touch or contaminate the absorbent part. Then, the patch was labeled prior to storage in plastic bags at −20 °C until analysis. Placebo sweat patches, which all tested negative for methylone by LC-MS/MS, were used as drug-free blank samples. 

### 4.4. Sample Preparation and Analyte Measurement

Each patch was fortified with 0.5 mL of 200 ng/mL of IS solution and allowed to dry at room temperature for 30 min. The outer adhesive part of the patch was removed, and the absorbent part was cut into small pieces and placed in a glass tube with 2 mL of methanol for 1 h. Then, the methanolic solution was transferred to another glass tube. Finally, 100 µL was transferred to an autosampler vial, prior to the injection of 1 µL into the HPLC-MS/MS system.

Methylone and its metabolites’ concentrations in sweat were determined through a 1290 Infinity II HPLC coupled to a 6470A triple-quadrupole mass spectrometer (Agilent Technologies, Palo Alto, CA, USA), with an electrospray ionization source set in positive mode (ESI+). The separation of the compounds was obtained through a Kinetics^®^ 2.6 µm Phenyl-Hexyl 100 × 2.1 mm column (Phenomenex^®^). Then, 0.1% formic acid in water and acetonitrile were used as mobile phases A and B, respectively, and the flow rate was set to 0.4 mL/min. The elution gradient consisted of an initial condition of 5% B, held for 1.0 min, gradually increased to 50% B within 2.0 min, then increased to 95% B within 4.0 min, and finally decreased to 5% B and held for 6.0 min. The total run time was 6 min. The autosampler and column oven temperatures were set to 10 °C and 37 °C, respectively.

The mass spectrometer operated in multiple reaction monitoring (MRM) acquisition mode, selecting two transitions for each analyte and IS, as validated in our previous study. 

Validation parameters are presented in Supplementary Material Table S1. Using the above-reported analytical method, the limits of detection were 1.5, 0.2 and 0.2 ng/patch and the limits of quantification were 5.0, 0.5 and 0.5 ng/patch for methylone, MDC and HMMC, respectively.

## 5. Conclusions

The analytical method proved to be suitable for the detection of these compounds in sweat, with good accuracy, precision, sensitivity and linearity. 

Sweat represented an unconventional matrix, allowing the detection of methylone and its metabolite MDC at a few hours and at 24 h after the administration of 50, 100, 150 and 200 mg doses. HMMC was not detectable at any time interval. Thus, as expected, the parent drug was the principal analyte detected in sweat samples. However, investigators should take into consideration that the concentration of a drug in sweat can be affected by several factors, such as the different perspiration rates, the volume of sweat and the pharmacological activity of methylone regarding the body temperature, resulting in high inter-subject variability. Sweat is a suitable matrix for methylone and its metabolites’ determination in clinical and toxicological studies, providing a concentration that reveals recent drug consumption.

## Figures and Tables

**Table 1:** Characteristics of the participants.

	Methylone 50 mg (*n* = 3)	Methylone 100 mg (*n* = 5)	Methylone 150 mg (*n* = 4)	Methylone 200 mg (*n* = 7)	Placebo (*n* = 14)
Age (years)	22.3 ± 0.6 (22–23)	22.7 ± 0.8 (22–24)	23.4 ± 0.9 (22–24)	24.0 ± 0.0 (24–24)	23.3 ± 0.9 (22–24)
Weight (kg)	69.7 ± 14.2 (60.4–86.0)	71.0 ± 12.3 (60.4–87.0)	71.9 ± 10.0 (61.9–87.0)	70.0 ± 3.9 (66.7–76.6)	70.2 ± 8.7 (60.4–87.0)
Height (cm)	178.4 ± 3.4 (175.0–181.8)	177.0 ± 2.8 (181.8–174.2)	176.9 ± 4.8 (172.8–185.0)	183.6 ± 9.8 (172.8–193.5)	180.4 ± 7.2 (172.8–193.5)
BMI (kg/m^2^)	21.9 ± 4.5 (18.3–27.0)	22.7 ± 3.9 (18.3–27.9)	23.1 ± 3.7 (19.4–27.9)	21.0 ± 3.3 (18.0–25.7)	21.7 ± 3.5 (18.0–27.9)

## Data Availability

Data are contained within the article.

## Supplementary Materials

The following supporting information can be downloaded at: https://www.mdpi.com/article/10.3390/ijms24087395/s1.
