# Supplementary material for: Sweat Testing for the Detection of Methylone after Controlled Administrations in Humans
Source: Int J Mol Sci. 2023 Apr 17;24(8):7395. doi: 10.3390/ijms24087395 (PMC10138602; doi:10.3390/ijms24087395)
Supplement: Supplementary file 1 [file ijms-24-07395-s001.zip › ijms-2324381-supplementary.pdf]

Supplementary Material

Table S1. Method validation parameters.

	Linear range (ng/patch)	r^2	p-value	$F_{crit95\%}$	LOD (ng/patch)	LOQ (ng/patch)	QC (ng/patch)			Accuracy (%)			Intra-day imprecision (CV%)			Inter-day imprecision (CV%)			Recovery (%)			Matrix effect (%)		
							**L**	**M**	**H**	**L**	**M**	**H**	**L**	**M**	**H**	**L**	**M**	**H**	**L**	**M**	**H**	**L**	**M**	**H**
Methylone	5-10000	0.9988	2.764	0.563	1.5	5.0	15	4000	8000	101.3	103.2	99.3	5.2	3.7	2.1	4.2	4.1	5.2	101.2	97.4	101.1	4.7	3.1	2.7
MDC	0.5-100	0.9996	0.546	2.675	0.2	0.5	1.5	40	80	94.8	97.4	98.8	0.7	2.7	5.2	1.7	2.8	2.1	98.1	97.9	96.2	0.5	-2.5	-0.5
HMMC	0.5-100	0.9999	0.741	0.908	0.2	0.5	1.5	40	80	108.1	94.8	107.2	1.3	2.1	2.9	3.3	7.1	3.8	103.3	99.3	101.4	-2.1	1.6	-6.1

Abbreviations: CV, coefficient of variation; H, high-quality control; HMMC, 4-hydroxy-3-methoxy-N-methylcathinone; L, low-quality control; LOD, limit of detection; LOQ, limit of quantification; M, medium-quality control; MDC, methylendioxycathinone; QC, quality control; r^2, correlation coefficient.
